# Tracing and Forecasting Metabolic Indices of Cancer Patients Using Patient-Specific Deep Learning Models

**DOI:** 10.3390/jpm12050742

**Published:** 2022-05-02

**Authors:** Jianguo Hou, Jun Deng, Chunyan Li, Qi Wang

**Affiliations:** 1 Beijing Computational Science Research Center, Beijing 100193, China; jghou@csrc.ac.cn; 2 Department of Therapeutic Radiology, Yale University School of Medicine, New Haven, CT 06510, USA; jun.deng@yale.edu; 3 Department of Mathematics, University of South Carolina, Columbia, SC 29208, USA; chunyan@email.sc.edu

**Keywords:** deep learning, dynamical systems, LSTM, metabolic panel, prediction, time series

## Abstract

We develop a patient-specific dynamical system model from the time series data of the cancer patient’s metabolic panel taken during the period of cancer treatment and recovery. The model consists of a pair of stacked long short-term memory (LSTM) recurrent neural networks and a fully connected neural network in each unit. It is intended to be used by physicians to trace back and look forward at the patient’s metabolic indices, to identify potential adverse events, and to make short-term predictions. When the model is used in making short-term predictions, the relative error in every index is less than 10% in the $L_{\infty }$ norm and less than 6.3% in the $L_{1}$ norm in the validation process. Once a master model is built, the patient-specific model can be calibrated through transfer learning. As an example, we obtain patient-specific models for four more cancer patients through transfer learning, which all exhibit reduced training time and a comparable level of accuracy. This study demonstrates that this modeling approach is reliable and can deliver clinically acceptable physiological models for tracking and forecasting patients’ metabolic indices.

## 1. Introduction

With advancements in medical research and practices, more and more cancer patients can live a long time after their cancer treatments, making the quality of life and toxicity management during and post-treatment the primary focus of healthcare providers and cancer patients. How to detect early health anomalies and predict potential adverse effects from some measurable biomarkers or signals for an individual cancer patient is of great importance in cancer patient care [1,2]. The latest Cancer Moonshot 2.0 initiative announced by President Biden aims at reducing the death rate of cancer by 50% in 25 years, which once again put a spotlight on the early detection, diagnosis, and management of cancer [3]. Developing the ability to track a patient’s health status and to monitor the evolution of the specific disease intelligently would provide an enormous benefit to both the patient and the healthcare provider, enabling faster responses to deal with adverse effects and more precise and effective treatments and interventions. With the longitudinally collected time-series data of the patient (e.g., metabolic panel, blood panel test, X-ray and CT scans, etc.) at multiple time points before, during, and after cancer treatments, it is becoming increasingly plausible to have an intelligent tool or device for continuous monitoring, tracking, and forecasting of cancer patients’ health status based on statistical, causal, and mechanistic modeling of patient phenotypes and various biomarkers in the time series data [1,2,4,5,6,7].

The concept of digital twins in healthcare is emerging as a promising platform to develop such a model/tool and gaining a great deal of traction in cancer research and, more broadly, the healthcare community lately [8,9]. The theme in digital twins is to develop a replicate of the targeted health state of the individual patient with an underlying disease based on the patient medical history (medical record, diagnosis, treatments, etc.), the evolutionary trajectory of the disease, and the current health status of the patient. The digital twin model will not only replicate the targeted patient’s state of health relevant to the underlying disease at any given historical time point, but also be able to make a future inference or prediction for the patient’s state of health. Retroceding the patient health state back in time is a distinctive feature that a digital twin has while any live human being does not. With the digital twin’s monitoring, tracking, and predictive capability, physicians can examine the past treatments virtually and explore various treatment pathways/strategies in the future by conducting virtual experiments to come up with an optimal treatment plan/strategy for any specific patient. With this, the digital twin can become a de facto intelligent aide to assist physicians in making decisions in their diagnostic processes and the design of future treatment plans, pathways, or recovery therapies. As a fundamental component in the digital twin model, a physiological module capable of time-reversal monitoring, tracking, and forward prediction of the metabolic panel or the blood panel is indispensable [10].

Recent advances in deep learning applications in healthcare have received a great deal of attention and achieved stunning results in many areas, especially in imaging processing. There have been numerous efforts on deep-learning-based diagnoses using imaging classification [11,12,13]. From an evolutionary perspective, any human body is a dynamical system sustained essentially by food and water intake, as well as air inhalation, while being bombarded incessantly by various external disturbances. Any portion of the human body such as the blood, metabolism, or an individual organ forms its own living subsystem, which perhaps is unavoidably coupled to the entire body’s dynamical system. By ignoring the coupling between the subsystem and the whole body, we assume there exists a self-sustained dynamical system such as the metabolic system in any human body. Any metabolic panel taken from a patient at a given time point would provide a glimpse of the state of the subsystem at that time. With sufficiently collected time series longitudinal data, one would be able to establish a fairly reasonable dynamical system model to describe the underlying dynamics within the subsystem. Such a dynamical system model would possess desired time reversibility in that one can use it to track the previous states in the past and to predict future events as well.

In this study, we aim to develop an AI-enabled deep learning model to monitor, track, and predict a cancer patient’s health status reflected through the metabolic panel from the patient medical records. It is expected to be used by physicians to predict the patient’s treatment outcome trajectory, to reduce the treatment-related adverse effects in the short term, and to improve the quality of life of the cancer patient in the long term. This dynamic model will be patient-specific and updated whenever new health data of the patient become available. A long-short-term memory (LSTM) recurrent neural network (RNN) with a new coupled cell architecture is devised and trained by machine learning based on the time series data in the metabolic panel of cancer patients treated at the Yale-New Haven Health System. We demonstrate how to develop the data-driven, patient-specific model from a set of selected indices from the metabolic panel taken from a cancer patient [14,15,16,17,18,19].

To demonstrate the model’s generalizability to other cancer patients, we recalibrated the model parameters against the metabolic data of the new patient using transfer learning. We believe this modeling approach provides a reliable technical framework for building the physiological module in any digital twin models in healthcare [20].

## 2. Materials and Methods

We first describe the patient data and data preprocessing used in machine learning and then detail the various LSTM models we developed in this study.

### 2.1. Data Acquisition and Preprocessing

With the institutional review board’s approval (Yale HIC#1604017609), five cancer patients aged 8 to 17 years old, who have received radiation treatments at Yale-New Haven Hospital between 2013 and 2021, were identified and their metabolic panel results were extracted from the EPIC electronic medical record (EMR) system per the HIPPA regulations. Nineteen metabolic indices were measured during each metabolic procedure for all the cancer patients: Glucose, BUN, Creatinine, BUN/Creatinine Ratio, Anion Gap, CO_2_, Chloride, Sodium, Potassium, Calcium, Total Protein, Albumin, Globulin, A/G Ratio, Aspartate Aminotransferase (AST), Alanine Aminotransferase (ALT), AST/ALT Ratio, Alkaline Phosphatase, and Total Bilirubin. A series of metabolic panel assessments was performed on each patient at multiple time points (ranging from 20 to 50 times) before, during, and after their cancer radiotherapy.

We selected 9 biomarkers/indices in the patients’ metabolic panel data with the minimal missing data rate as the time series dataset to establish the time-dependent discrete dynamical system. These indices include Glucose, BUN, Creatinine, Anion Gap, CO_2_, Chloride, Sodium, Potassium, and Calcium. The chosen data for the first patient, whom we name the original patient, was acquired on 42 consecutive days spanning the period from 10 May 2013 to 14 May 2014. We filled the few missing entries in the dataset by using the mean of the two nearest neighbors, i.e., if index $x_{i}$ is missing, we “assign it” the value of $\frac{x_{i-1}+x_{i+1}}{2}$. The missing data involve the Creatinine index on 26 June 2013 and the Calcium index on 2 January 2014, 3 January 2014, and 4 January 2014, respectively. After making up the missing data points, we computed the mean μ and standard deviation σ of the entire 42 data points for every index. If an index value was larger than μ+2σ or less than μ−2σ, we replaced it by μ+2σ or μ−2σ, respectively, in the dataset to rule out the so-called outlier effect.

Since the clinical data were acquired at non-uniform time intervals, to derive an approximate discrete dynamical system to describe the time series using recurrent neural networks, we needed to generate more data with equal time intervals that included the initial 42 9-dimension/index clinical dataset. We used the first-order linear interpolation to to obtain 739 9-dimensional data vectors, in which adjacent data points are separated from each other by 0.5 days. The correlation coefficients of the 9 indices at the 739 data points are tabulated in Table 1. It shows that no pair of indices in the dataset was highly correlated with a correlation coefficient ≥80%. Hence, we will built the discrete dynamical system using all indices as the input variables of the dynamical system. We chose the LSTM RNN as the framework to build the discrete dynamical system from the preprocessed dataset.

### 2.2. One-Step Predictive LSTM Model

**Figure 1:**
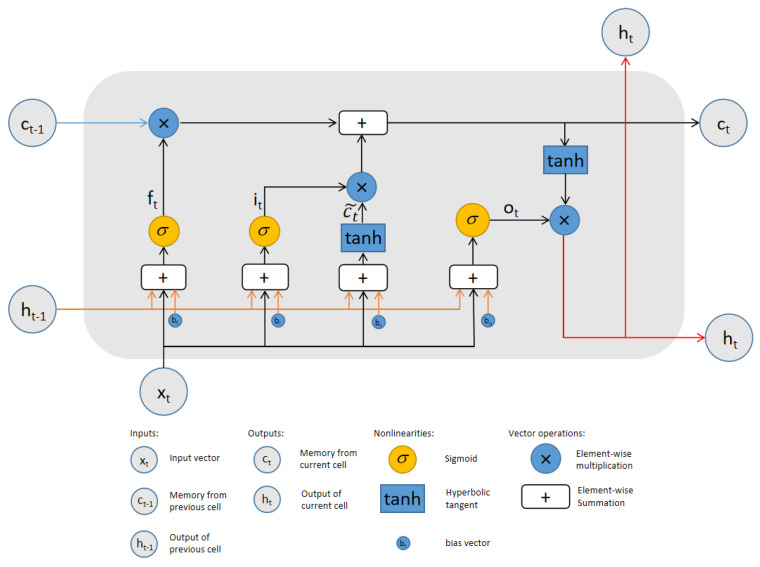
Schematics of an LSTM cell.

An LSTM model provides a versatile recurrent neural network architecture with a great deal of flexibility to overcome the gradient vanishing and explosion problems inherent in the conventional recurrent neural networks, while capturing transient dynamics underlying the time series data [19,21,22]. When designing its architecture for our applications, we paid close attention to the input and output data structure to make sure they could describe the underlying time-dependent dynamics since the input and output of an LSTM model do not need to be in the same data format or structure as those in the original metabolic dataset. The general structure of an LSTM cell is shown in Figure 1, where $x_{t}$ is the input to the recurrent neural network cell, $c_{t}$ is the state in the LSTM to enhance the memory effect, and $h_{t}$ is the output of the cell.

A generic LSTM cell is given by the following mathematical formula, where ⨀ indicates the Hadamard product.

(1) $$\begin{array}{c} \begin{array}{c} f_{t}=\sigma \left(W_{f}\cdot \left[h_{t-1},x_{t}\right]+b_{f}\right),\\ i_{t}=\sigma \left(W_{i}\cdot \left[h_{t-1},x_{t}\right]+b_{i}\right),\\ {\tilde{c}}_{t}=\tanh\left(W_{c}\cdot \left[h_{t-1},x_{t}\right]+b_{c}\right),\\ c_{t}=f_{t}⨀c_{t-1}+i_{t}*{\tilde{c}}_{t},\\ o_{t}=\sigma \left(W_{o}\left[h_{t-1},x_{t}\right]+b_{o}\right),\\ h_{t}=o_{t}⨀\tanh\left(c_{t}\right),\\ y_{t}=\sigma \left(W^{\prime }h_{t}\right). \end{array} \end{array}$$

In the LSTM cell, $f_{t}$ is called the forget gate and $i_{t}$ the input gate. We multiply the cell state $c_{t-1}$ by the forget gate $f_{t}$ to control the propagation of the previous information. Then, we add $i_{t}⨀{\tilde{c}}_{t}$ to update the current cell state $c_{t}$. In this process, we combine the previous information with the new information to obtain the final current cell state. Then, we calculate $h_{t}$ and output $y_{t}$ from $c_{t}$ and the previous hidden state $h_{t-1}$.

**Figure 2:**
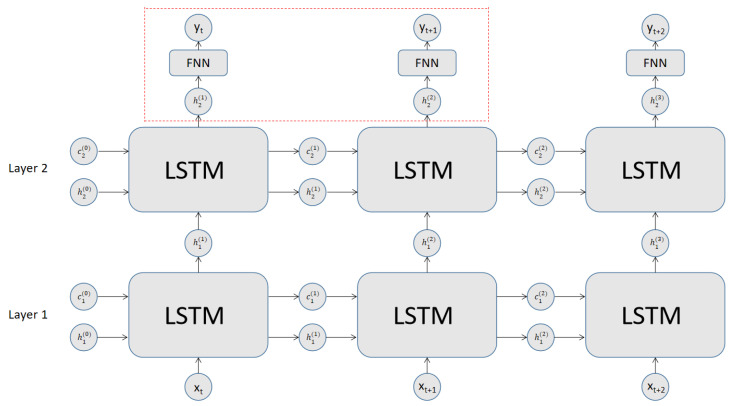
Schematics of an RNN cell consisting of a pair of stacked LSTM cells and a fully connected neural network at the output layer. The part in the rectangular box bounded by the dashed lines is optional.

In our design of the model, we used a stacked LSTM architecture coupled with a fully connected neural network for each cell. Figure 2 is a schematic portrait of the stacked LSTM architecture that we adopted in our LSTM model.

The pair of LSTM cells stacked in series and connected to a fully connected output neural network was aimed to achieve a better memory effect. In fact, we can stack more LSTM cells intercalated with fully connected neural networks to form a more complex composite LSTM cell, in which the final output layer taking output $h_{t}$ as the input is a fully connected neural network.

**Figure 3:**
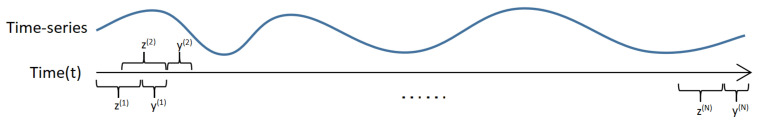
Schematics of the input and output data format/structure.

For time series data $\left[x_{1},x_{2},\cdots ,x_{n}\right]$, where $x_{i}$ represents the *i*-th 9-dimensional data vector from the original dataset, and a given time step T>0, we concatenate the input 9-dimensional metabolic index data points into a large vector $z^{\left(i\right)}$ = $\left[x_{i},x_{i+1},\cdots ,x_{i+T-1}\right]$ to define our input to the stacked LSTM cell and define the output of the LSTM cell as $y^{\left(i\right)}$ = ${\hat{x}}_{i+T}$. The data structure is depicted in Figure 3. The number of total new input–output data pairs $\left(z^{\left(i\right)},y^{\left(i\right)}\right)$ in the new data structure is N=n−T, given as follows

(2) $$\begin{array}{c} \begin{array}{c} z^{\left(1\right)}=\left[x_{1},x_{2},\cdots ,x_{T}\right],y^{\left(1\right)}={\hat{x}}_{T+1},\\ z^{\left(2\right)}=\left[x_{2},x_{3},\cdots ,x_{T+1}\right],y^{\left(2\right)}={\hat{x}}_{T+2},\\ \cdots \\ z^{\left(N\right)}=\left[x_{N},x_{N+1},\cdots ,x_{T+N-1}\right],y^{\left(N\right)}={\hat{x}}_{T+N}. \end{array} \end{array}$$

We designed the LSTM with time step T, input vector $z^{\left(i\right)}$ and output $y^{\left(i\right)},i=1,\cdots ,N$. By applying the LSTM model through the concatenated dataset $(z^{\left(i\right)},y^{\left(i\right)}),i=1,\cdots ,N$ as an RNN, we predict the next output using the previous T input vectors from the original dataset. We thus name this the one-step predictive LSTM model.

The loss function in the model is defined by

(3) $$\begin{array}{c} Loss=\frac{1}{M}\sum_{i=1}^{M}\frac{\|{\hat{x}}_{T+i}-x_{T+i}{\|}_{2}^{2}}{9}, \end{array}$$

where *M* is the number of output data vectors in the batch of data.

For the 739 9-dimensional data points in the original time series, we first divided them into the training set and test set in a 9:1 ratio sequentially. With this division, the number of data points in the training set and test set was 665 and 74, respectively. For the data sets, we carried out the zero-mean standardization in each index. Namely, for the *j*-th index, we computed the mean $\mu^{j}$ and unbiased standard deviation $\sigma^{j}$ of the 665 data points in the training set. Then, we standardized the training data and test data as follows:

(4) $$\begin{array}{c} {\tilde{x}}_{i}^{j}=\frac{x_{i}^{j}-\mu^{j}}{\sigma^{j}}, \end{array}$$

where *i* represents the *i*-th data point and *j* represents the vector’s *j*-th entry. Then, we used the standardized training data for the model. The number of input and output pairs of the LSTM model is N=739−T, and the data pairs are given by $(z^{\left(i\right)},y^{\left(i\right)}),i=1,\cdots ,N$. The number of training data pairs is 665−T given by $\left(z^{\left(1\right)},y^{\left(1\right)}\right)$ to $\left(z^{\left(665-T\right)},y^{\left(665-T\right)}\right)$. The number of the data pairs used for the test is 74 ranging from $\left(z^{\left(666-T\right)},y^{\left(666-T\right)}\right)$ to $\left(z^{\left(739-T\right)},y^{\left(739-T\right)}\right)$.

First, we set a loss tolerance ϵ in the training of the neural network. If the training loss was lower than ϵ, we saved the current model parameters and applied the model to the test data. After a prescribed number of epochs, we chose the model that gave the best outcome over the test data as our chosen model. Through an extensive experimentation, we adopted the following hyperparameters for the LSTM model as tabulated in Table 2.

The time step is one of the key hyperparameters in the LSTM model. We trained the model with respect to time step T=1,⋯,10. The final value of the step is determined by the one that gives the best performance. To evaluate the performance of the model, we define the following metrics:

(5) $$\begin{array}{c} \begin{array}{c} RMSE=\sqrt{\frac{1}{n}\sum_{i=1}^{n}{\left(y_{i}-{\hat{y}}_{i}\right)}^{2}},\\ NSE=1-\frac{\sum_{i=1}^{n}{\left(y_{i}-{\hat{y}}_{i}\right)}^{2}}{\sum_{i=1}^{n}{\left(y_{i}-\mu \right)}^{2}},\;\mu =\frac{1}{n}\sum_{i=1}^{n}y_{i},\\ MAPE=relative\;L_{1}\;error=\frac{1}{n}\sum_{i=1}^{n}\frac{\left|y_{i}-{\hat{y}}_{i}\right|}{y_{i}},\\ relative\;L_{\infty }\;error=\max_{1\leq i\leq n}\frac{\left|y_{i}-{\hat{y}}_{i}\right|}{\left|y_{i}\right|}. \end{array} \end{array}$$

Note that the LSTM is a versatile recurrent neural network (RNN); there is a great deal of flexibility in its design, especially in its input and output data structures. We showcase some other LSTM designs here and compare their performance with the previous one.

### 2.3. Input-Stacked LSTM Model

Given the versatility of an LSTM design, we can stack the input and output vectors to form larger input and output vectors. For example, when stack number S=3, we first stack data vectors as follows: $X_{i}={\left[x_{i},x_{i+1},x_{i+2}\right]}^{T}$, where $x_{i}$ is the *i*-th 9-dimensional data vector. Then, the input and output data pairs are defined in the following forms:

(6) $$\begin{array}{c} \begin{array}{c} z^{\left(1\right)}=\left[X_{1},X_{2},\cdots ,X_{T}\right],y^{\left(1\right)}=\left[{\hat{x}}_{1+S},{\hat{x}}_{2+S},\cdots ,{\hat{x}}_{T+S}\right],\\ z^{\left(2\right)}=\left[X_{2},X_{3},\cdots ,X_{T+1}\right],y^{\left(2\right)}=\left[{\hat{x}}_{2+S},{\hat{x}}_{3+S},\cdots ,{\hat{x}}_{T+1+S}\right],\\ \cdots \\ z^{\left(N\right)}=\left[X_{N},X_{N+1},\cdots ,X_{T+N-1}\right],y^{\left(N\right)}=\left[{\hat{x}}_{N+S},{\hat{x}}_{N+1+S},\cdots ,{\hat{x}}_{T+N-1+S}\right]. \end{array} \end{array}$$

The loss for a batch of output data is defined by:

(7) $$\begin{array}{c} Loss=\frac{1}{N}\sum_{t=1}^{N}\sum_{p=1}^{T}\frac{\|{\hat{x}}_{p+t-1+S}-x_{p+t-1+S}{\|}_{2}^{2}}{9\cdot T}. \end{array}$$

### 2.4. Multistep Predictive Model

In the models discussed above, prediction step L=1. Next, we examine how well the model performs if we extend the one-step predictive model to multiple steps L>1 by revising the design. Here, the input–output data pairs are defined as follows:

(8) $$\begin{array}{c} \begin{array}{c} z^{\left(1\right)}=\left[x_{1},x_{2},\cdots ,x_{T}\right],y^{\left(1\right)}=\left[{\hat{x}}_{T+1},{\hat{x}}_{T+2},\cdots ,{\hat{x}}_{T+L}\right],\\ z^{\left(2\right)}=\left[x_{2},x_{3},\cdots ,x_{T+1}\right],y^{\left(2\right)}=\left[{\hat{x}}_{T+2},{\hat{x}}_{T+3},\cdots ,{\hat{x}}_{T+L+1}\right],\\ \cdots \\ z^{\left(N\right)}=\left[x_{N},x_{N+1},\cdots ,x_{T+N-1}\right],y^{\left(N\right)}=\left[{\hat{x}}_{T+N},{\hat{x}}_{T+N+1},\cdots ,{\hat{x}}_{T+N+L-1}\right]. \end{array} \end{array}$$

The hyperparameters used are again the ones in Table 2.

## 3. Results

We present the results obtained using the LSTM models for the original patient alluded to in the previous section firstly. Then, we showcase the results of the patient-specific model for each of the other four patients obtained from transfer learning.

### 3.1. One-Step Prediction

We considered both the $relative\;L_{1}\;error$ and the $relative\;L_{\infty }\;error$ of the nine indices when choosing the best time step *T* for the LSTM RNN. The errors of the LSTM model with T=1,⋯,10, when applied to the test set, are listed in Table 3. It follows from Table 3 that the LSTM with time step T=2 gives the best one-step prediction. We note that the dimensional results are converted from the standardized, dimensionless quantities as follows:

(9) $$\begin{array}{c} {\hat{y}}_{i}^{j}={\bar{y}}_{i}^{j}\cdot \sigma^{j}+\mu^{j}, \end{array}$$

where $\bar{y}$ is the dimensionless quantity used in the neural network model.

**Figure 4:**
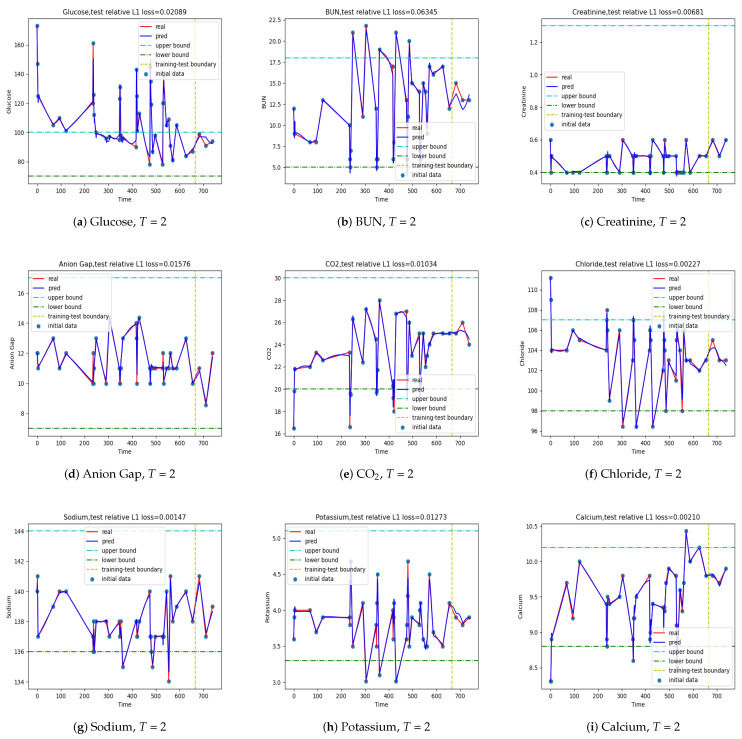
Output $\left\{{\hat{y}}_{i},i=1,\cdots ,\right\}$ of the LSTM with time step T=2. The values used in training are plotted on the left of the vertical line, while the predicted ones are on the right. The largest $L_{1}$ error is about 1.5%, and the largest $L_{\infty }$ error is about 9%.

The results of the one-step predictive model with T=2 are depicted in Figure 4, where the maximum relative error is about 9% and the average relative error is about 1.5%. These results are clinically acceptable. Table 4 lists the relative errors of each index for the LSTM model with T=2. Except for the largest relative error at about 9%, all others are significantly less than 9%, showing that the one-step predictive model is fairly accurate. When we varied the time step *T*, we noticed that the LSTM model in fact gave the smallest average error at time step T=1; however, its absolute error was larger than the model with T=2. As *T* increased, the average error increased as well, and so did the absolute error. There was an exception at T=6 though, where the absolute error was much smaller than the model at T=5 and T=7. As a result, we chose T=2 as the hyperparameter of the LSTM model in making one-step predictions.

### 3.2. Input-Stacked LSTM Model

For time step T=1,⋯,5, respectively, we varied stack number *S* from one to four to examine the effect of the stack number on the outcome of the LSTM model. The other hyperparameters of the input-stacked LSTM are given in Table 2. We show the minimum training loss after 300 epochs in Table 5 with respect to different stack numbers. The results in Table 5 show that the fitting capacity of the stacked LSTM improved as the number of stacks increased. This is because, when the number of stacks increases, more historical information from the input is added to improve the training loss. For the generalization error of the trained model over the stacked test dataset, we computed the average relative $L_{1}$ error of the nine indices for a set of selected stack numbers. The results are shown in Table 5 as well. Notice that the generalization error of the model increased as the stack number increased, indicating increased overfitting in the training of the LSTM model as *S* increases since the input of the training set and testing set can be quite different after all. This study indicated that using the input-stacked LSTM may not gain any advantages in improving generalization errors over the non-stacked one alluded to earlier, at least for the dataset we used.

### 3.3. Multistep Prediction

**Figure 5:**
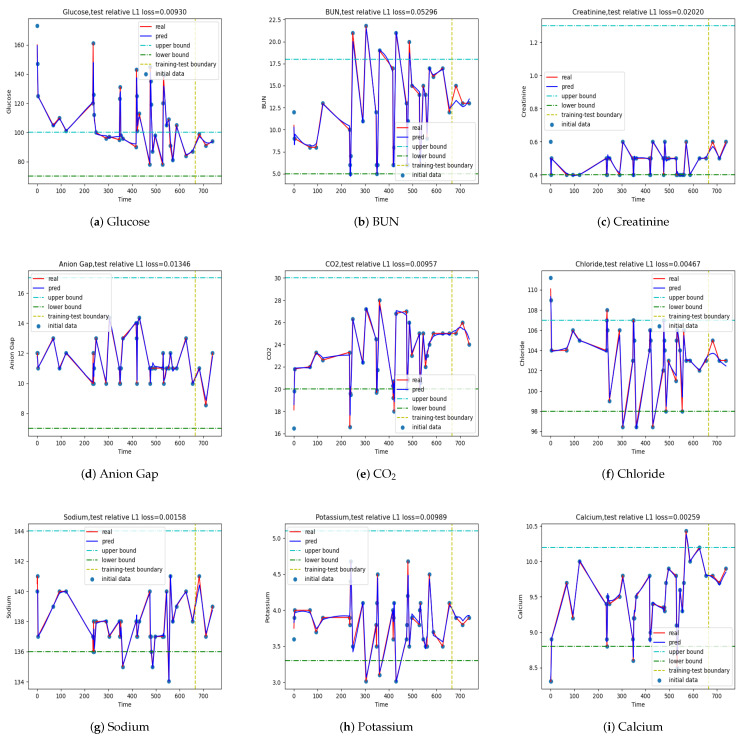
Output $\left\{{\hat{x}}_{T+L+i-1},i=1,\cdots ,\right\}$ at T=1, L=1. The ones used in training are plotted on the left of the vertical line and the predicted ones on the right.

**Figure 6:**
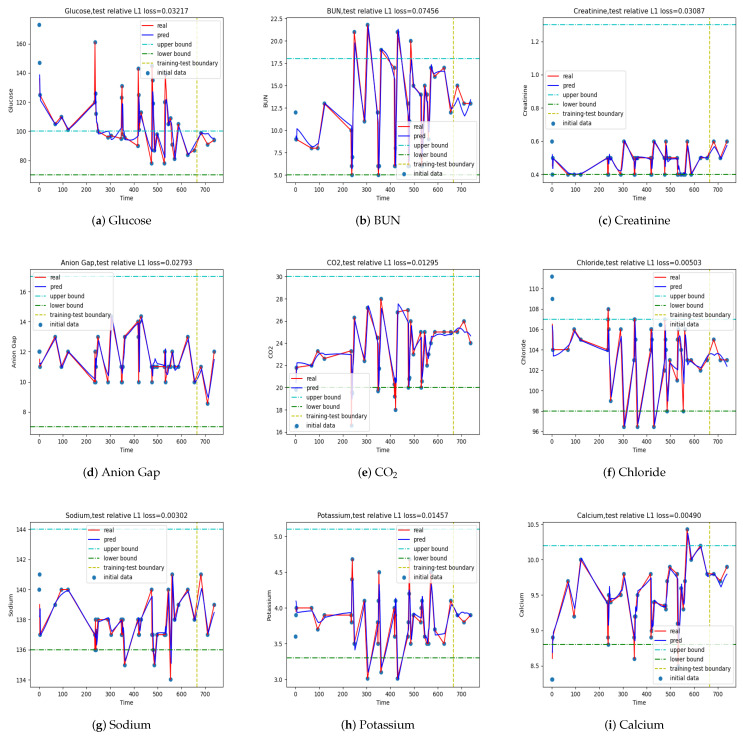
Output $\left\{{\hat{x}}_{T+L+i-1},i=1,\cdots ,\right\}$ at T=1, L=3. The ones used in training are plotted on the left of the vertical line and the predicted ones on the right.

We tabulate the results in the largest $L_{1}$ relative error and the largest $relative\;L_{\infty }\;error$ for L=1,2,3,4 with respect to T=1,2,3,4,5 in Table 6, respectively. From the results in the table, we concluded that time step T=1 performed the best in this LSTM model, in which the average error was in general not amplified much; however, the relative $L_{\infty }$ norm grew significantly as *L* increased, especially for large *L*. The results of the LSTM with T=1 and different prediction step L=1,3 are depicted in Figure 5 and Figure 6, respectively, as two examples.

In principle, we can use the LSTM model with L>1 for multistep predictions. However, the predictive power deteriorates rapidly while *L* increases. For instance, the largest $relative\;L_{\infty }\;error$ was about 10.8 % at L=3, and it increased to 14.7% at L=4. The average errors in the $L_{1}$ norm were much smaller than those in the $L_{\infty }$ norm. The largest $L_{1}$ error at L=4 was less than 4.2% in the study.

### 3.4. Model Comparison

In one-step predictions, we found that the input-stacked LSTM model improved the model’s fitting capacity at the expense of decreased computational efficiency, although the fitting capacity of the one-step predicative LSTM model was sufficient for the current problem without using stacked input data. However, while fitting capacity improved, the generalization error in the input-stacked LSTM model may deteriorate as the number of the predicative steps increases. Therefore, our assessment of the two LSTM models is that the one-step predicative LSTM model without stacked input data is simpler and perhaps the better model to use for the given dataset.

In multistep predictions, we directly added multiple output steps. After an extensive search for the hyperparameters, we can extend the prediction steps to L=3 under the restriction that the largest relative $L_{\infty }$ loss among all indices is around 10%. We saw relative errors larger than 10% for steps beyond L=3 and, therefore, would advise against using the model beyond L=3 for the given dataset.

### 3.5. Transfer Learning of LSTM Models to Fit Other Patients

Given the patient-specific LSTM models presented above, we next discuss how to apply the models to other patients through transfer learning. In transfer learning, the hyperparameters of the deep neural network model are kept so that the training of the deep neural network is much more efficient in terms of epochs. The patient-specific models for the four other patients through transfer learning showed similar accuracy in short-term predictions, demonstrating the effectiveness and reliability of this approach. Specifically, we employed transfer learning to retrain the model parameters (weight and biases) to fit the same set of indices from the metabolic panel of the other four cancer patients, coded respectively as ESK, MS, PH, and SCC, while retaining the hyperparameters of the LSTM model.

#### 3.5.1. One-Step Prediction

For the four additional patients, we fixed the hyperparameters of the LSTM model given in Table 2 and then retrained the weight and biases of the LSTM using the parameters in the already trained model as the initial guess. The patient-specific model for each patient was trained in a much-reduced number of epochs. The corresponding relative $L_{1}$ and $L_{\infty }$ errors for all four patient-specific LSTM models are tabulated in Table 7. The models for patients ESK and SCC trained especially well with relative errors better than the ones from the model for the original patient. The “worse” and the “worst” case were given by the models for patient PH and MS, respectively. If we examine the model for PH closely, we notice that the $L_{1}$ error in the model was consistently less than 8.6% for all indices, while the $L_{\infty }$ error of BUN was about 17.5%, Anion Gap was about 11.22%, and others were all less than 9.4%; the NSE value corresponding to BUN and Anion Gap was 0.612 and 0.045, respectively, indicating the fitting was performed reasonably well, although they were not accurately reflected by the relative errors. Analogously, we noticed that the $L_{1}$ error for BUN in the model for patient MS was 32%, while the $L_{\infty }$ was over 100%. This is because the relative error metric used in this case involves a small number in the denominator, where an absolute error metric is perhaps more accurate than the relative error metric. The actual NSE value for BUN in this model was 71.42%. Therefore, the fitting result is not that bad.

**Figure 7:**
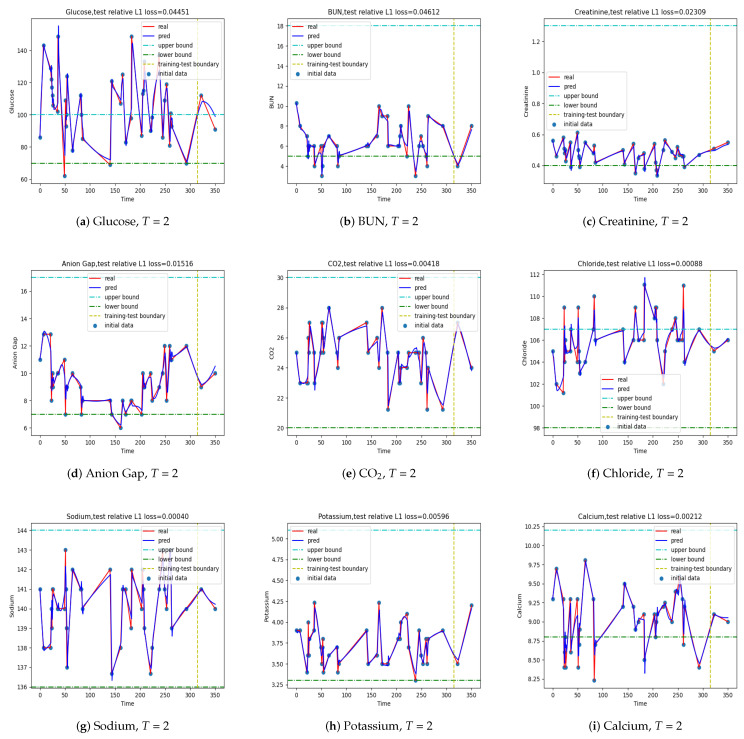
The outputs of the one-step predictive LSTM model for patient ESK with time step T=2. The values on the right of the vertical line are predicted.

**Figure A1:**
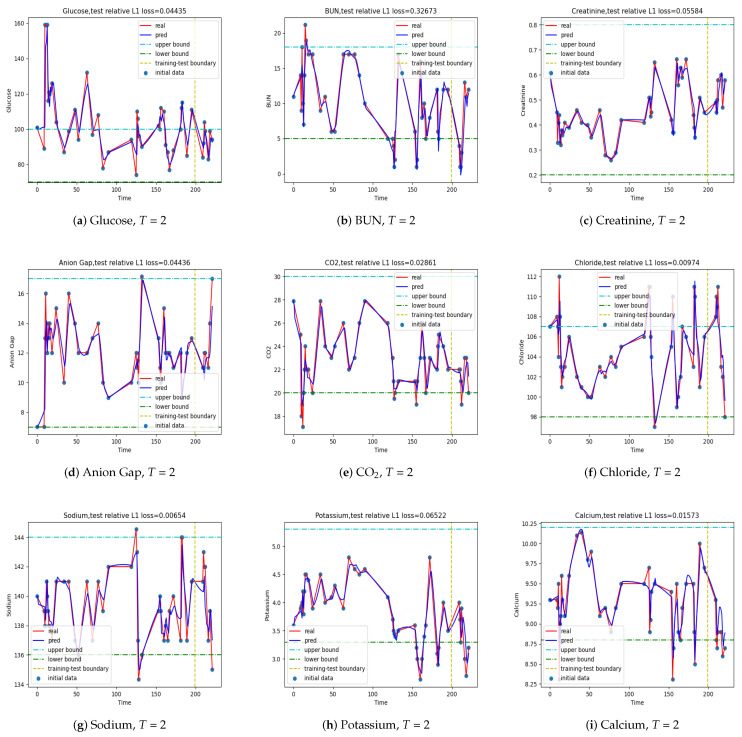
The one-step prediction of the LSTM model for patient MS with time step T=2.

**Figure A2:**
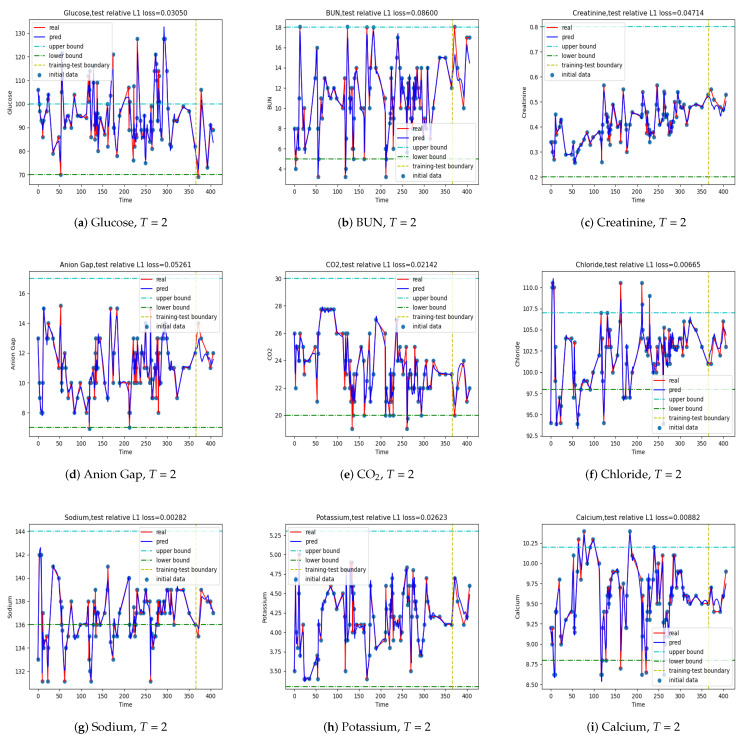
The one-step prediction of the LSTM model for patient PH with time step T=2.

**Figure A3:**
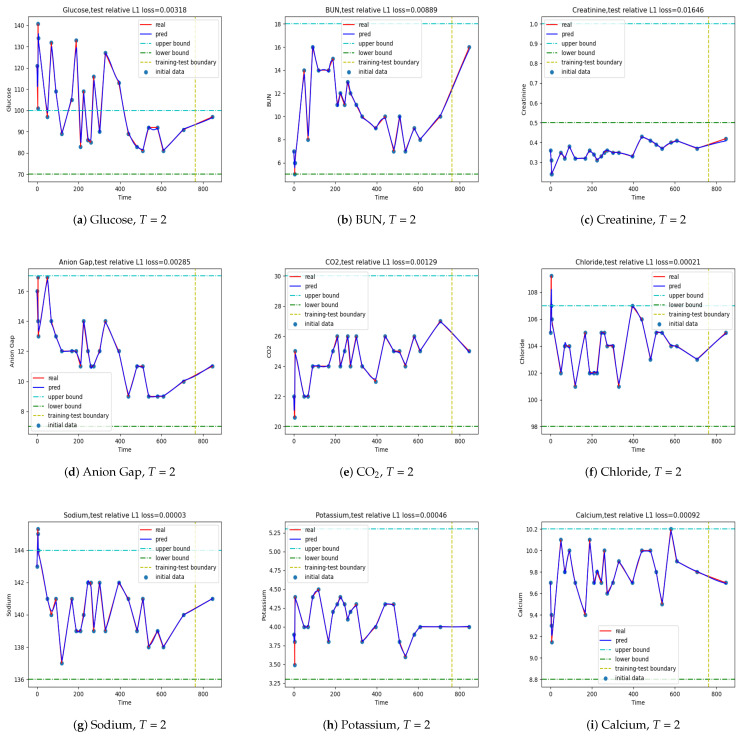
The one-step prediction of the LSTM model for patient SCC with time step T=2.

The input and output data and the one-step prediction for patients ESK, PH, SM, and SCC are plotted in Figure 7, Figure A1, Figure A2 and Figure A3, respectively. The specific models for patients ESK and SCC made better predictions than the original one. If we examine the absolute error, the model for patient MS was no worse than the one for patient ESK. We depict Figure 7 in the text here and put Figure A1, Figure A2 and Figure A3 in the Appendix A.

#### 3.5.2. Multistep Prediction

**Figure 8:**
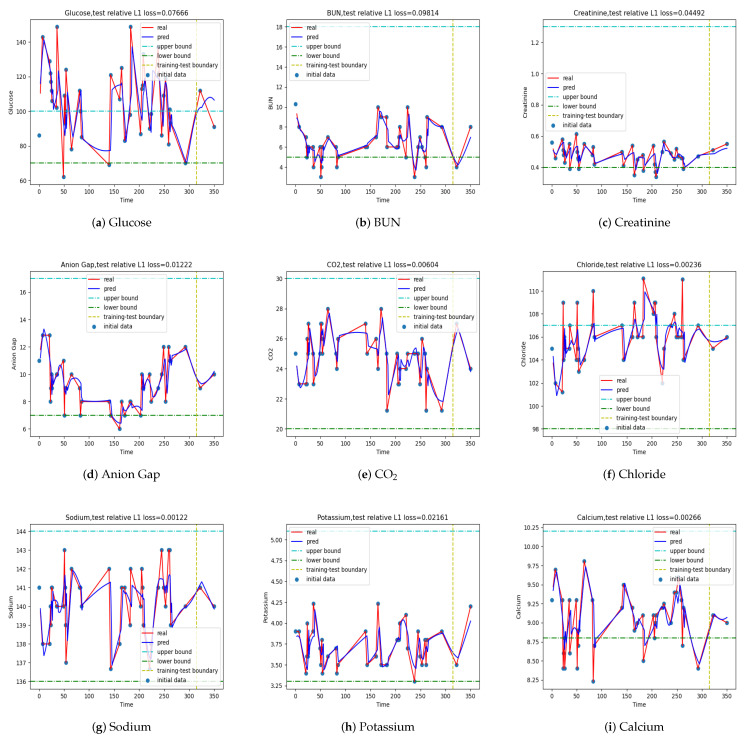
Multi-step predictions of the LSTM model for patient ESK at T=1, L=3. The values on the right of the vertical line are predicted.

**Figure 9:**
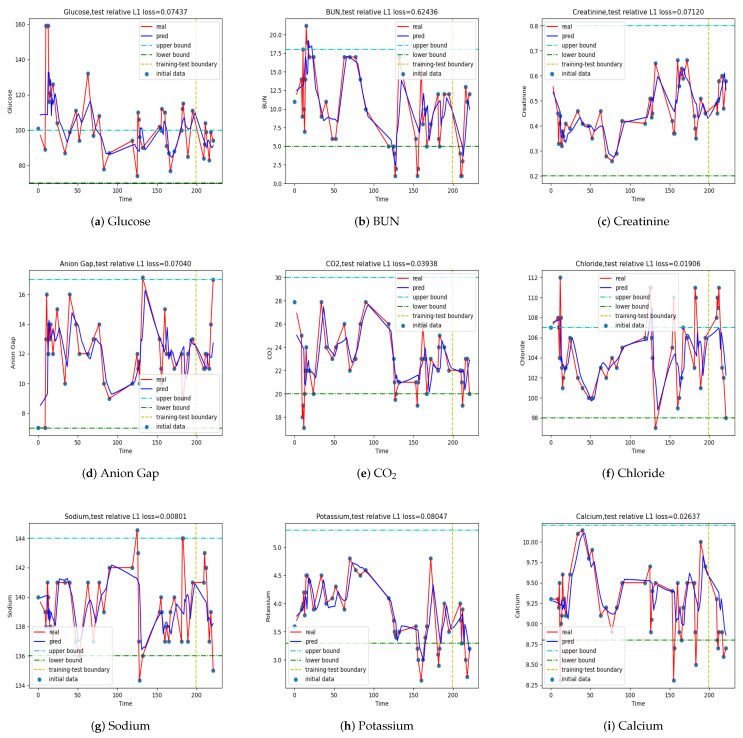
Multistep predictions of the LSTM model for patient MS at T=1, L=3.

**Figure A4:**
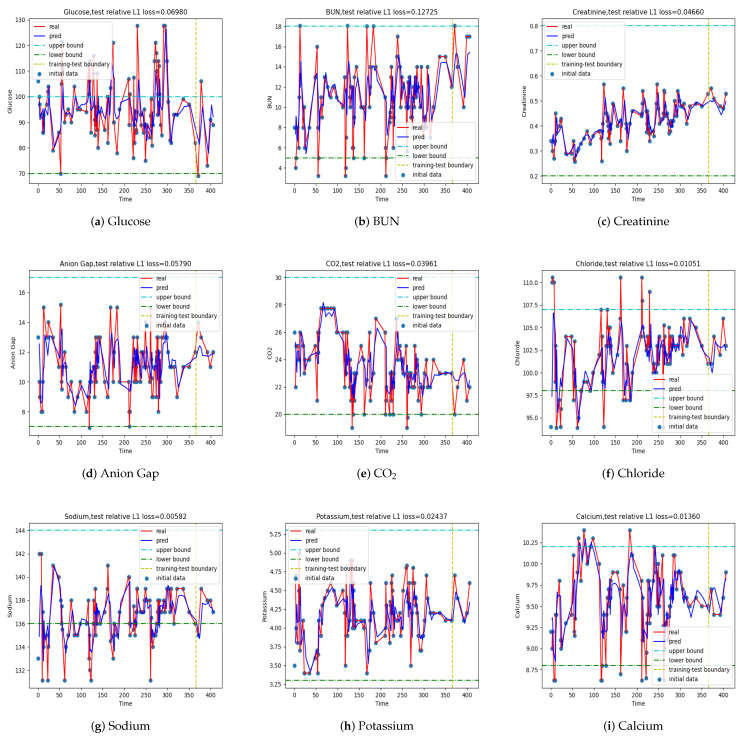
The multistep prediction of the LSTM model for patient PH with T=1, L=3.

**Figure A5:**
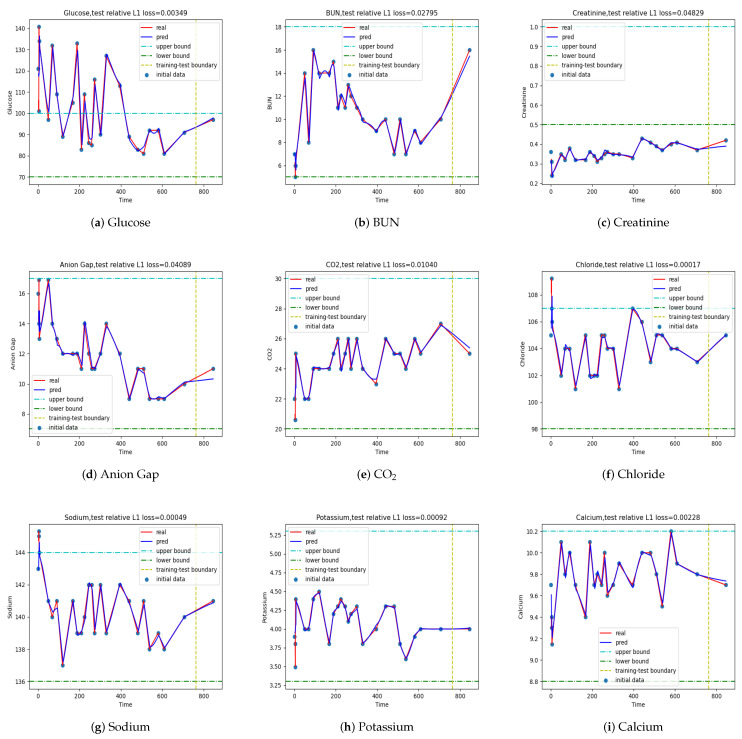
The multistep prediction of the model for patient SCC with T=1, L=3.

Then, we checked the performance of the models in making multistep predictions, where the hyperparameters of the LSTM models are given in Table 2 and the prediction step L=3. We plot model outputs for time step L=3 in Figure 8, Figure 9, Figure A4, and Figure A5, respectively. The corresponding relative errors are tabulated in Table 8. The results are comparable to those in the one-step prediction if we take the relative error as the evaluation criterion: we can achieve good multistep predictions on patients ESK, PH, and SCC, while we cannot predict as well on patient MS. However, the prediction made by the model for patient MS performed reasonably well in BUN, as shown in Figure 9, if other assessment metrics besides the relative errors are taken into account. We show Figure 8 and Figure 9 here and put Figure A4 and Figure A5 in the Appendix A.

## 4. Discussion

A human being is a complex dynamical system. Hence, the indices in the patient’s metabolic panel follow an intrinsic evolutionary path in time, which is undoubtedly influenced by many health factors within the human body. As a first step toward establishing the digital twin model for the patient, it would be reasonable to assume that the indices in the metabolic panel form a self-consistent dynamical system. Under this assumption, we embarked on a journey to build a discrete dynamical system model to approximate the underlying dynamics of the patient based on an LSTM RNN. The model leverages the historical data acquired in time series to infer the future behavior of the indices in the metabolic panel in time. The rationale for adopting the LSTM architecture to build the discrete dynamical system model is because of its versatility in the structure and functional design to mimic a discrete dynamical system using deep neural networks. We note that we have had some successful experience with continuous dynamical system modeling of time series data in the past for albumin dynamics coupled with several other biomarkers using neural ordinary differential equations [7]. We tried the previous approach for the given datasets. Unfortunately, that approach did not produce a desirable result for the time series data in the metabolic panel that we studied.

In this study, we were limited by the availability of accurate raw data in all indices. Therefore, we only applied the modeling framework to a subset of the indices in the metabolic panel to showcase the effectiveness of the modeling approach and the usefulness of the resulting models. We believe we can readily extend this to all indices in the metabolic panel should a complete dataset be available. In the model developed for the first cancer patient, the one-step prediction amounted to about 12 h or 1/2 day. In both the absolute value metric ($L_{\infty }$ norm) and the average $L_{1}$ norm, the relative error was less than 10%. These error ranges are in general acceptable clinically. When the five models were applied to make one-step predictions, the trend was generally predicted correctly and the errors were in acceptable ranges. The model is expected to give physicians a reasonable short-term prediction with a correct trend and a reasonably close value to assist their decision-making in treatment planning, medical history tracking, and monitoring.

From all the numerical experiments conducted, we noticed that the multistep predictions of the models were in general less accurate than those in the one-step predictions. This is because the model we used relies heavily on adequate and accurate data, but there is inevitably noise in the data collected from the patients over time and the amount of data from each patient is relatively small, limited by the current medical practices and regulations. For the medical data, how to reduce the data noise and improve the robustness of the data-driven modeling is still a challenging problem to be solved. We noticed that there were some existing predictive models for other medical issues in the literature that could obtain a smaller relative $L_{1}$ error (e.g., lower than 1%) [23,24]. The temporal fluctuations in the metabolic panel data we acquired were more significant. We should not expect the same level of error in the outcome of the predictive model. In addition, we added the feasibility study of transfer learning of the deep learning model to other patients of the same disease and established the corresponding clinically acceptable results. If we want to apply the patient-specific model in the real-world scenario, the model must have the ability to be transferred to new patients.

Moving forward, we plan to apply the developed LSTM RNN framework to predict the risks of stroke and heart disease for individuals based on their time series metabolic biomarkers. This future work is clinically important, as heart diseases and strokes are the #1 and #5 causes of death in the United States, respectively [25]. That our LSTM model can accurately predict the personal metabolic variations some period ahead is a critically important feature of this model as the early warning and prevention procedures could largely reduce the risk of heart attack and stroke, hence saving lives and improving the quality of life for millions of people worldwide. We will report the results of this new development in our future work.

In addition, we will explore the possibility of developing separate dynamical models based on an individual’s other blood test results, a procedure often performed in the clinic to evaluate one’s overall health status and detect a wide range of disorders such as anemia, infection, and leukemia. Similar to the comprehensive metabolic panel (CMP), there is a series of complete blood count (CBC) results registered for individual patients in the electronic medical record (EMR) system. However, the biomarkers are different with different correlations between them; hence, different machine learning algorithms may be needed to better describe the dynamics of health status using these time series CBC data. Furthermore, we will test if the higher dimensions of patient data by combining CMP and CBC biomarkers could lead to better prediction over a 12 h’ early warning time. All these efforts will eventually lead to the ultimate physiological digital twin module we would like to develop for a patient, not only to replicate the patient physiological state from the past to now, but also to make reasonable inferences for the near future.

Limited by sparsity in the time points when the longitudinal data were collected, continued assessment of the model on a larger cohort of cancer patients and assessment of model robustness on augmented longitudinal data collected at refined time points are necessary. The lack of statistical details in the data collection points in the training dataset may also limit the inference capability of the model. All these are challenging issues that need further refined analyses.

## 5. Conclusions

We developed a discrete dynamical system model describing the time-dependent dynamics of the metabolic panel of a cancer patient using the LSTM recurrent neural network architecture. The patient-specific model can be used to make short-term predictions in one step with relative errors consistently less than 10% in the absolute value metric and much less than 10% in an average sense. It can be applied to make multistep predictions with a slightly elevated error level (i.e., relative error less than 11% in three steps and 15% in four steps). Using four additional cancer patients’ metabolic panels, we show that the patient-specific LSTM model can be retrained through transfer learning. This modeling platform has great potential for identifying potential dynamical features in the metabolic panel of cancer patients and patients of other prominent diseases, and thereby serves as an important module in the more general digital twin for the cancer patient.

## Figures and Tables

**Table 1:** Correlation coefficients of the 9 metabolic indices over the interpolated data points.

	Glucose	BUN	Creatine	Anion Gap	CO_2_	Chloride	Sodium	Potassium	Calcium
Glucose	1								
BUN	−0.566336	1							
Creatine	−0.283779	0.548764	1						
Anion Gap	−0.0871597	0.435452	0.282975	1					
CO_2_	−0.744225	0.757975	0.351549	0.0799233	1				
Chloride	0.602712	−0.784478	−0.382908	−0.368508	−0.792248	1			
Sodium	−0.0771287	0.0227023	−0.0117806	0.138286	0.0923114	0.373784	1		
Potassium	0.0807286	−0.543226	−0.012367	−0.422068	−0.47736	0.52685	−0.0705765	1	
Calcium	−0.754321	0.575592	0.301983	0.118504	0.613964	−0.506214	0.0840671	−0.0404592	1

**Table 2:** Hyperparameters of the LSTM model.

	Train–Test Proportion	Batch Size	Learning Rate	Optimizer	Error Tolerance ϵ	LSTM Direction	Time Steps
initial patient one-step prediction	9:1	16	0.001	Adam	0.01	unidirectional	1–10
initial patient input-stacked LSTM	9:1	16	0.001	Adam	0.05	unidirectional	1–5
initial patient multistep prediction	9:1	16	0.001	Adam	1	unidirectional	1–5
new patients’ one-step prediction	9:1	16	0.001	Adam	0.01	unidirectional	2
new patients’ multistep prediction	9:1	16	0.001	Adam	1	unidirectional	2

**Table 3:** The $Relative\;L_{1}\;Error$ and $Relative\;L_{\infty }\;Error$ of the LSTM model at different time steps *T*.

Time Step *T*	*Relative* $L_{1}$ *Error*	*Relative* $L_{\infty }$ *Error*
*T* = 1	0.01380	0.1141
*T* = 2	0.01509	0.08914
*T* = 3	0.01509	0.1052
*T* = 4	0.01811	0.1264
*T* = 5	0.02027	0.1249
*T* = 6	0.02072	0.09490
*T* = 7	0.02074	0.1327
*T* = 8	0.02159	0.1360
*T* = 9	0.02245	0.1395
*T* = 10	0.02189	0.1388

**Table 4:** Relative errors for all 9 indices of the metabolic panel in the LSTM model with T=2.

Index	*Relative* $L_{1}$ *Error*	*Relative* $L_{\infty }$ *Error*
Glucose	0.02088	0.06424
BUN	0.06344	0.08914
Creatinine	0.006806	0.01754
Anion Gap	0.01575	0.03049
CO_2_	0.01034	0.03068
Chloride	0.002267	0.007395
Sodium	0.001465	0.003298
Potassium	0.01273	0.02356
Calcium	0.002098	0.005678

**Table 5:** The minimum training loss and average $relative\;L_{1}\;error$ at different stack numbers S.

**Minimum Training Loss**	**Stack 1**	**Stack 2**	**Stack 3**	**Stack 4**
*T* = 1	0.03369	0.02792	0.02101	0.01742
*T* = 2	0.02616	0.02240	0.01712	0.01454
*T* = 3	0.02282	0.01954	0.01548	0.01297
*T* = 4	0.02092	0.01770	0.01437	0.01178
*T* = 5	0.01956	0.01636	0.01334	0.01091
$\mathrm{relative}\;L_{1}\;\mathrm{error}$	**Stack 1**	**Stack 2**	**Stack 3**	**Stack 4**
*T* = 1	0.02154	0.02312	0.02484	0.02427
*T* = 2	0.01939	0.02326	0.02068	0.02108
*T* = 3	0.01652	0.02211	0.02225	0.02312
*T* = 4	0.01708	0.02277	0.02408	0.02556
*T* = 5	0.01664	0.02310	0.02575	0.02792

**Table 6:** The *relative* $L_{1}$*error* and the *relative* $L_{\infty }$ *error* in multistep predictions.

$L_{1}$ ***Error***	***L* = 1**	***L* = 2**	***L* = 3**	***L* = 4**
*T* = 1	0.01380	0.01790	0.02288	0.02905
*T* = 2	0.01431	0.02465	0.02930	0.03480
*T* = 3	0.01345	0.02896	0.03721	0.04095
*T* = 4	0.01251	0.03118	0.04020	0.04209
*T* = 5	0.01217	0.03127	0.04137	0.04174
$\mathrm{relative}\;L_{\infty }\;\mathrm{error}$	***L* = 1**	***L* = 2**	***L* = 3**	***L* = 4**
*T* = 1	0.1141	0.09797	0.1077	0.1464
*T* = 2	0.0976	0.2665	0.2750	0.2685
*T* = 3	0.1039	0.3177	0.3478	0.3584
*T* = 4	0.1048	0.3893	0.3520	0.3313
*T* = 5	0.09047	0.4020	0.3737	0.2987

**Table 7:** Relative $L_{1}$ and $L_{\infty }$ errors for all 9 indices of the patients.

Index	ESK $L_{1}$	MS $L_{1}$	PH $L_{1}$	SCC $L_{1}$	ESK $L_{\infty }$	MS $L_{\infty }$	PH $L_{\infty }$	SCC $L_{\infty }$
Glucose	0.04450	0.04435	0.03049	0.003183	0.08778	0.1067	0.08891	0.003952
BUN	0.04611	0.3267	0.08600	0.008891	0.06270	2.777	0.1750	0.01438
Creatinine	0.02308	0.05583	0.04714	0.01645	0.03092	0.2203	0.09399	0.02750
Anion Gap	0.01515	0.04435	0.05260	0.002854	0.05069	0.1528	0.1122	0.0065
CO_2_	0.004182	0.02860	0.02141	0.001285	0.01014	0.09882	0.05195	0.002946
Chloride	0.0008751	0.009741	0.006651	0.0002089	0.002677	0.03172	0.05195	0.0007556
Sodium	0.0003999	0.00654	0.002817	$3.474\times 10^{-5}$	0.001599	0.01926	0.008932	0.0001211
Potassium	0.005959	0.06521	0.02623	0.0004617	0.01528	0.1765	0.04095	0.001250
Calcium	0.002123	0.01572	0.008822	0.0009209	0.005822	0.05646	0.01663	0.001075

**Table 8:** Relative $L_{1}$ and $L_{\infty }$ errors for all 9 indices of the patients at L=3.

Index	ESK $L_{1}$	MS $L_{1}$	PH $L_{1}$	SCC $L_{1}$	ESK $L_{\infty }$	MS $L_{\infty }$	PH $L_{\infty }$	SCC $L_{\infty }$
Glucose	0.07665	0.07436	0.06979	0.003486	0.1691	0.1259	0.2092	0.008344
BUN	0.09813	0.6243	0.1272	0.02795	0.1337	4.712	0.2211	0.03364
Creatinine	0.04492	0.07119	0.04659	0.04829	0.05324	0.2566	0.09157	0.07156
Anion Gap	0.01222	0.07039	0.05790	0.04089	0.04159	0.2514	0.1319	0.06099
CO_2_	0.006038	0.03937	0.03960	0.01040	0.02352	0.1511	0.1280	0.01529
Chloride	0.002357	0.01906	0.01050	0.0001742	0.005595	0.04345	0.02913	0.0002961
Sodium	0.001221	0.008014	0.005817	0.0004871	0.002988	0.02370	0.01990	0.0009125
Potassium	0.02160	0.08046	0.02436	0.0009167	0.04192	0.2703	0.07769	0.002582
Calcium	0.002662	0.02636	0.01359	0.002278	0.007707	0.07977	0.03365	0.003785

## Data Availability

The data presented in this study are available on request from the corresponding author. The data are not publicly available due to privacy issues.

## Appendix A

### Appendix A.1. Other Plots of the Transfer Learning

The corresponding output plots of the other patients are shown below.

### Appendix A.2. Statistical Difference

The mean and standard deviation of the raw data from the five patients, including training and testing data, are tabulated in Table A1 and Table A2, respectively. The mean values of the patient data were relatively close, but the variances were different, indicating data fluctuations among different patients.

**Table A1:** Mean values of the indices of in the patients’ data.

Index	Initial	ESK	MS	PH	SCC
Glucose	101.375	99.5275	98.604	94.1602	98.7027
BUN	13.473	6.46195	10.3432	11.5713	10.827
Creatinine	0.481732	0.477585	0.451914	0.423641	0.366111
Anion Gap	11.6233	9.29698	11.9656	11.1393	11.3348
CO_2_	23.9059	24.8989	23.5392	23.8356	24.8449
Chloride	102.826	105.838	104.09	101.897	103.923
Sodium	138.207	140.124	139.6	136.927	140.11
Potassium	3.77487	3.72149	3.94011	4.14017	4.06711
Calcium	9.60211	9.05112	9.40042	9.63124	9.79586

**Table A2:** Standard deviations in the data of the five patients’.

Index	Initial	ESK	MS	PH	SCC
Glucose	12.2582	19.0009	13.4126	10.3282	13.3031
BUN	3.62326	1.42737	4.13605	3.0001	2.40213
Creatinine	0.0565706	0.0468106	0.0914002	0.072239	0.0360429
Anion Gap	1.14982	1.64735	2.19155	1.64426	1.78242
CO_2_	1.80619	1.57407	2.39049	2.0276	1.16956
Chloride	2.34336	1.75886	2.93231	3.07383	1.23467
Sodium	1.23496	1.35068	2.02357	1.98712	1.26889
Potassium	0.268589	0.184193	0.480437	0.337178	0.189848
Calcium	0.324299	0.300906	0.34867	0.370416	0.15704

### Appendix A.3. Performance Measures of All One-Step LSTM Models

We define the absolute mean error as

(A1) $$\begin{array}{c} MAE=\frac{1}{n}\sum_{i=1}^{n}\left|y_{i}-{\hat{y}}_{i}\right|. \end{array}$$

The detailed error estimates of the patient-specific, one-step prediction LSTM models are tabulated in the following five tables with time step T=2. From the tables, we find the model works well with the MAPE evaluation criterion. Because of the intrinsic problem in the formula of the NSE, the results of the NSE can be bad when the data are close to a constant. Hence, we focus on the results given by the MAPE.

**Table A3:** Errors in other metrics of the one-step prediction of the original patient.

Index	RMSE	MAE	MAPE	NSE
Glucose	2.515	1.946	0.02088	−0.2631
BUN	0.9432	0.8726	0.06344	−0.9996
Creatinine	0.004453	0.003744	0.006806	0.9747
Anion Gap	0.1792	0.1617	0.01575	0.9555
CO_2_	0.3339	0.2619	0.01034	0.5067
Chloride	0.3051	0.2359	0.002267	0.8091
Sodium	0.2374	0.2038	0.001465	0.9576
Potassium	0.05616	0.04940	0.01273	0.02123
Calcium	0.02376	0.02049	0.002098	0.7503

**Table A4:** Errors in other metrics of the one-step prediction of patient ESK.

Index	RMSE	MAE	MAPE	NSE
Glucose	5.022	4.422	0.04450	0.3181
BUN	0.2929	0.2707	0.04611	0.9428
Creatinine	0.01230	0.01212	0.02308	0.3026
Anion Gap	0.1951	0.1459	0.01515	0.5312
CO_2_	0.1237	0.1074	0.004182	0.9798
Chloride	0.1171	0.09219	0.0008751	0.8365
Sodium	0.08001	0.05614	0.0003999	0.9317
Potassium	0.02549	0.02263	0.005959	0.9867
Calcium	0.02429	0.01916	0.002123	0.5892

**Table A5:** Errors in other metrics of the one-step prediction of patient MS.

Index	RMSE	MAE	MAPE	NSE
Glucose	4.791	4.129	0.04435	0.3112
BUN	1.842	1.296	0.3267	0.7141
Creatinine	0.04124	0.02867	0.05583	0.3251
Anion Gap	0.9817	0.6057	0.04435	0.6150
CO_2_	0.8399	0.6080	0.02860	0.2224
Chloride	1.411	1.022	0.009741	0.8205
Sodium	1.207	0.9144	0.006542	0.6265
Potassium	0.2668	0.2183	0.06521	0.5498
Calcium	0.1938	0.1392	0.01572	0.6879

**Table A6:** Errors in other metrics of the one-step prediction of patient PH.

Index	RMSE	MAE	MAPE	NSE
Glucose	3.475	2.651	0.03049	0.8609
BUN	1.485	1.262	0.08600	0.6123
Creatinine	0.02752	0.02417	0.04714	−0.2638
Anion Gap	0.8056	0.6734	0.05260	0.04500
CO_2_	0.5665	0.4698	0.02141	0.7005
Chloride	0.8055	0.6843	0.006651	0.6286
Sodium	0.4940	0.3872	0.002817	0.8139
Potassium	0.1200	0.1133	0.02623	0.5025
Calcium	0.09582	0.08412	0.008822	0.5527

**Table A7:** Errors in other metrics of the one-step prediction of patient SCC.

Index	RMSE	MAE	MAPE	NSE
Glucose	0.3143	0.3038	0.003183	0.9106
BUN	0.1327	0.1277	0.008891	0.9840
Creatinine	0.007148	0.006713	0.01645	0.3344
Anion Gap	0.03527	0.03060	0.002854	0.9594
CO_2_	0.03899	0.03266	0.001285	0.9876
Chloride	0.02992	0.02187	0.0002089	0.9927
Sodium	0.007090	0.004883	$3.47\times 10^{-5}$	0.9983
Potassium	0.002221	0.001847	0.0004617	$-2.36\times 10^{26}$
Calcium	0.009061	0.008960	0.0009209	0.7326
